# Vulto-van Silfhout-de Vries syndrome caused by *de novo* variants of *DEAF1* gene: a case report and literature review

**DOI:** 10.3389/fneur.2023.1251467

**Published:** 2023-11-24

**Authors:** Hui Zhu, Shuyao Zhu, Qiong Jiang, Ying Pang, Yu Huang, Yan Chen, Ting Hou, Wenxin Deng, Xingyu Liu, Lan Zeng, Ai Chen, Jin Wang, Zemin Luo

**Affiliations:** ^1^Department of Pediatrics, Sichuan Provincial Maternity and Child Health Care Hospital, Chengdu, China; ^2^Department of Neonatology, Sichuan Provincial Maternity and Child Health Care Hospital, Chengdu, China; ^3^Department of Medical Genetics and Prenatal Diagnosis, Sichuan Provincial Maternity and Child Health Care Hospital, Chengdu, China

**Keywords:** Vulto-van Silfhout-de Vries syndrome, dominant mental retardation-24, *DEAF1*, *de novo*, intellectual disability, behavioral abnormalities

## Abstract

Vulto-van Silfhout-de Vries syndrome (VSVS; MIM 615828) is an extremely rare autosomal dominant disorder with unknown incidence. It is always caused by *de novo* heterozygous pathogenic variants in the *DEAF1* gene, which encodes deformed epidermal autoregulatory factor-1 homology. VSVS is characterized by mild to severe intellectual disability (ID) and/or global developmental delay (GDD), seriously limited language expression, behavioral abnormalities, somnipathy, and reduced pain sensitivity. In this study, we present a Chinese boy with moderate GDD and ID, severe expressive language impairment, behavioral issues, autism spectrum disorder (ASD), sleeping dysfunction, high pain threshold, generalized seizures, imbalanced gait, and recurrent respiratory infections as clinical features. A *de novo* heterozygous pathogenic missense variant was found in the 5th exon of *DEAF1* gene, NM_021008.4 c.782G>C (p. Arg261Pro) variant by whole exome sequencing (WES). c.782G>C had not been previously reported in genomic databases and literature. According to the ACMG criteria, this missense variant was considered to be “Likely Pathogenic”. We diagnosed the boy with VSVS both genetically and clinically. At a follow-up of 2.1 years, his seizures were well controlled after valproic acid therapy. In addition, the child’s recurrent respiratory infections improved at 3.5 years of age, which has not been reported in previous individuals. Maybe the recurrent respiratory infections like sleep problems reported in the literature are not permanent but may improve naturally over time. The literature review showed that there were 35 individuals with 28 different *de novo* pathogenic variants of *DEAF1*-related VSVS. These variants were mostly missense and the clinical manifestations were similar to our patient. Our study expands the genotypic and phenotypic profiles of *de novo DEAF1*.

## 1 Introduction

Vulto-van Silfhout-de Vries syndrome (VSVS; MIM 615828), also known as autosomal dominant mental retardation-24 (MRD24), is an autosomal dominant disorder caused by *de novo* heterozygous pathogenic variant in *DEAF1* gene (1). The major clinical phenotypes of VSVS include different levels (mild to severe) of intellectual disability (ID) and/or global developmental delay (GDD), serious language limitations, motor retardation, autism spectrum disorders (ASD), problems with behaving, somnipathy, and increased pain threshold. It is fairly rare with only 11 articles having described the disease and has an unknown incidence (1–11).

Herein, we report a Chinese male child with a novel *de novo* pathogenic missense variant in the *DEAF1* gene, namely, c.782G>C (p. Arg261Pro), that was confirmed by using whole-exome sequencing (WES) and Sanger sequencing verification. The phenotype of our patient was basically consistent with those reported in the literature. This report expands the known range of VSVS due to *de novo* pathogenic variants of the *DEAF1* gene. Our patient recurrent respiratory infections are not permanent. The phenomenon has not been described in previously reported cases. In addition, to summarize the clinical manifestations and genotypes, we also reviewed the VSVS cases reported in literature worldwide. We aim to raise clinicians’ knowledge and understanding of VSVS so that the disease gradually becomes no longer rare in the future.

## 2 Case presentation

A 3-year-old boy was admitted to the neurological in-patient department with ID, speech delay, and seizures in April 2021. He was born at term by cesarean section. His birth weight and length were 3,200 g and 48 cm, respectively. His non-consanguineous parents were healthy and were pregnant for the first time. There was no history of prenatal exposure to alcohol, drugs, or medications.

He presented GDD noticed since babyhood, but his development revealed no regression. He crawled at 8 months, began to grow baby teeth and was sitting at 1 year old, and could stand on his own by 1.6 years old. He could walk at the age of 2, and his gait was ataxic. He started speaking in bi-syllables (baba and mama) at 2.3 years of age, but his language ability was not improved at 3 years. He could not feed himself at 3 years old. The Gesell Developmental Scale indicated a developmental quotient score of 46, and the Standford-Binet Intelligence Scale indicated a score of 40 defined as moderate ID. He manifested insomnia after birth, and sleep disturbance disappeared when he was 4 years old. The child had a high pain threshold and the following abnormal behavioral manifestations: poor eye contact, restricted and repetitive behavior, fascination, attention deficit hyperactivity disorder (ADHD), irritability, aggressive behavior, and context-inappropriate laughter. From birth to date, he had recurrent respiratory tract infections characterized by an average of approximately once a month of upper respiratory tract infection or bronchopneumonia. Gastrointestinal infections occurred two to three rounds per year. There were no deformities of the face, limbs, and spine. His vision and hearing were normal. Muscle tone and strength were normal for four limbs. His first seizure occurred at 2.9 years of age, and by 3 years of age, he had had five rounds of seizures, and his seizure was generalized tonic–clonic.

Given the presence of poor eye contact, stereotyped behavior, fascination, ID, poor expressive speech, behavioral abnormalities, epilepsy, sleep dysfunction, and motor delay, a diagnosis of autism spectrum disorder and comorbid neurodevelopmental disorders (ASD-NDDs) was considered. Thus, blood samples of the child and his parents were collected for genetic analysis by WES.

**Figure 1:**
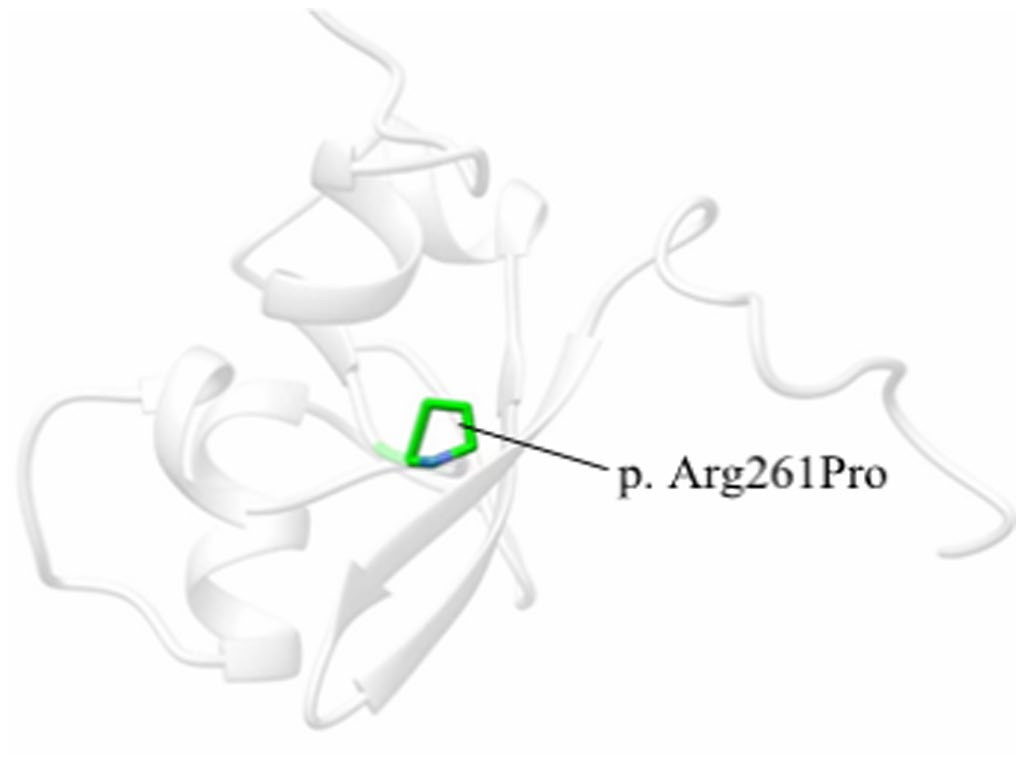
One 3D model of the SAND domain in this study based on the homology study of the solution structure of the human Sp100b SAND domain by heteronuclear NMR (PDBe 1h5p). The amino acid substitution is in green.

Ethical approval was provided by the institutional ethics committee. After informed consent was given, EDTA blood samples were obtained from the boy and his parents. WES was conducted and the methods have been described in detail in previous studies (12). PolyPhen-2, SIFT, Provean, Mutation Taster, and Mutation Assessor were used to predict whether the detected variant was damaging or causing disease. Based on the homology study of the solution structure of the human Sp100b and the SAND domain by heteronuclear NMR (PDBe 1h5p), the PDB structure of the SAND domain was modeled by phyre2 server software and modified by UCSF chimera software (Figure 1). The result of the variant was interpreted by the American College of Medical Genetics and Genomics (ACMG) guidelines (Genet Med. 2015) (13).

**Figure 2:**
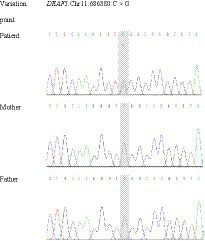
Sanger sequencing showing a heterozygous variant in DEAF1: the patient has a heterozygous pathogenic variant c.782G>C in exon 5, which is not present in either of the parents, suggesting the *de novo* occurrence of the variation.

The results of Trio-WES revealed a *de novo* heterozygous missense variant (NM_021008.4: c.782G>C, p. Arg261Pro) in the fifth exon of *DEAF1* gene, which was confirmed by Sanger sequencing (Figure 2). The variant was predicted to be damaging/disease-causing by PolyPhen-2 (score = 1), SIFT (score = 0.009), Provean (score = −5.29), Mutation Taster, and Mutation Assessor. The variant also affects evolutionary conserved amino acids within the *DEAF1* SAND domain. According to ACMG (13), the missense variant was classified as “Likely Pathogenic” with PS2 + PM2_Supporting + PP3. This pathogenic variant was not reported in 1000 Genomes, ExAC, gnomAD, ClinVar Databases, and previous literature. Certainly, at present, the pathogenic variant data of our patient has been deposited into the ClinVar bank and the accession number is VCV002444491.1.

Based on the clinical manifestations and genetic sequencing results, we diagnosed the boy with VSVS. His seizures were under treatment with valproic acid monotherapy that was started at 3 years of age and were well-controlled during the 2.1 years of follow-up. We followed up every 1–3 months by phone, WeChat chat, and clinic attendance. Every 3 months, there had to be an outpatient follow-up, detailed medical history, and physical examination to better record the child’s growth, development, and disease evolution. During the follow-up, we also found that the child’s recurrent respiratory infections disappeared at 3.5 years of age. Now, he is 5.1 years of age and is improving in his socio-adaptive skills, such as feeding himself.

## 3 Literature search

A systematic literature search in PubMed and OMIM was performed. MeSH and title or abstract were used for all eligible studies that mainly focused on the *DEAF1* variants in VSVS. The research strategy was as follows: “*DEAF1*” AND (“Vulto-van Silfhout-de Vries syndrome” or “autosomal dominant mental retardation-24” or “intellectual disability with speech impairment and behavioral problems”). Data from all eligible studies were analyzed and discussed by two reviewers. Detailed results are available in the “Discussion” section.

## 4 Discussion

VSVS is a relatively new and extremely rare autosomal dominant intellectual disability disease that has been discovered in the past 13 years, with few reports since the first case description by Vissers and his colleagues in 2010 (10). In 2014, Vulto-van Silfhout et al. (8) certified *de novo* heterozygous variations in *DEAF1* leading to VSVS in humans. The *DEAF1* gene (MIM 602635) encodes deformed epidermal autoregulatory factor-1 homology, which contains 12 exons and encodes 565 amino acids. This gene is highly expressed in brain cells, especially during the extremely young fetal development stage (8). As a transcriptional activator and repressor, *DEAF1* makes a vast difference in affecting the expression of diverse genes (2, 14–16). The main clinical manifestations of VSVS include different levels of GDD / ID, serious expressive language disorder, behavioral problems, autistic behaviors, somnipathy, and increased pain threshold (2).

We searched the world documentation previously reported from the first description in 2010 to March 2023. By March 2023, only 35 individuals, including adults and children, had been reported that carried *de novo* pathogenic variants of the *DEAF1* gene (1–11). Among the 35 cases, the median age was 7.1 years (range 1.3–38 years), with 14 women and 21 men. The major clinical phenotypes are summarized in Table 1. Different levels of ID / GDD and behavioral issues were described in all cases (35/35; 100%). Of those persons with behavioral abnormalities, the commonest was ASD (30/34; 88.2%). Severe expressive language barriers were presented in most persons (33/34; 97.1%), including 14 persons with speech absence and 19 cases with speech limitation. Lag of motor development and sleeping trouble were also usual at 84.8% (28/33) and 83.3% (25/30), respectively. In particular, it needs to be mentioned that sleep–wake disorders usually begin after birth and improve as the child grows. Reduced pain sensitivity was reported in 21 persons (21/26; 80.8%). Seizures were found in 21 cases (21/27; 77.8%), and the most common was generalized (18/27; 66.7%). Twenty-two individuals had abnormal gait (22/30; 73.3%) that incorporated gait ataxia (7/30; 23.3%) and other walking patterns (18/30, 60%) of broad-based and imbalanced gait or a tip-toe gait. Relatively less common clinical manifestations and those that are neglected and not described in detail include recurrent infections, mild facial deformities, and feeding difficulties (2). The major clinical features of our patient were similar phenotypes with previously described VSVS cases. Nevertheless, what calls for special attention is that the boy’s recurrent respiratory infections improved naturally at 3.5 years old, which has not been reported in the previous literature. We speculate that the recurrent respiratory infections in VSVS which are not permanent but are similar to previously reported sleep problems may improve naturally over time. Anyway, recurrent infections may be worth further studying in the future.

**Table 1:** Phenotype of patients with pathogenic *de novo* variants of *DEAF1* that have been previously reported.

Clinical features	Total (*N* = 35)
Gender	14F: 21 M
Median age (range)	7.1 years (1.3–38 years)
**Growth**	
Microcephaly	0/28 (0%)
Macrocephaly	4/28 (14.3%)
**Psychomotor development**	
DD/ID	35/35 (100%)
Motor delay	28/33 (84.8%)
Expressive speech	33/34 (97.1%)
Developmental regression	15/30 (50%)
Behavioral abnormalities	35/35 (100%)
Poor eye contact	20/28 (71.4%)
Autism	30/34 (88.2%)
Mood swings	25/28 (89.3%)
Fascinations	19/26 (73.1%)
Aggressive behavior	16/26 (61.5%)
Other behavioral problems	18/24 (75%)
**Neurologic abnormalities**	
Seizures	21/27 (77.8%)
Generalized seizures	18/27 (66.7%)
Partial seizures	2/27 (7.4%)
Unknown	1/27 (3.7%)
Abnormal walking pattern	22/30 (73.3%)
Gait ataxia	7/30 (23.3%)
Other walking problems	18/30 (60%)
High pain threshold	21/26 (80.8%)
Sleep disturbance	25/30 (83.3%)
Brain MRI abnormalities	8/24 (33.3%)
**Other abnormalities**	
Feeding difficulties	13/27 (48.1%)
Constipation	18/20 (90%)
Recurrent infections	18/27 (66.7%)

Seizures were observed in 77.8 percent of previously presented VSVS individuals. The epilepsies caused by *de novo* in *DEAF1* genes were predominantly generalized. Regarding the curative effect observation of antiepileptic drugs (AEDs) treatment, hitherto, there have been only two studies with small samples or case series, but their conclusions were opposite. One study included six individuals with VSVS, and the result showed almost all generalized persons (5/6; 83.3%) had no epileptic seizures by the use of valproic acid therapy (1). In contrast, the other study included 10 individuals, which revealed that epilepsies were not well controlled in the overwhelming majority of individuals (9/10;90%) (2). However, we found that only one individual was given valproic acid and other individuals were given other AEDs in the study of Nabais Sa et al. (2). Owing to the seizures of VSVS being ordinarily generalized, valproic acid could be the first choice in AEDs (1). Chen et al. (1) speculated that the interaction between valproic acid and the *DEAF1* gene may inhibit neuronal excitation. The difference in the results of the two studies on antiepileptic efficacy may be attributed to the function of different pathogenic variants in the *DEAF1* gene leading to different responses to AEDs (1, 2). Additionally, because the individuals in the two studies were treated with different AEDs, an accurate assessment was not available. Furthermore, the number of cases in the current relevant studies is small, making it difficult to draw more precise conclusions. Therefore, every case of VSVS is important and should be described in detail. Our patient had a generalized seizure. His seizures were well controlled by taking valproic acid monotherapy, and there were no seizures during his 2.1 years of follow-up. Of course, we will continue to follow up on this child for a long time.

*DEAF1* includes the following five functional domains: SAND, ZnF, NLS, NES, and MYND (2). Twenty-eight *de novo* pathogenic variants of the *DEAF1* gene were reported in the literature including 23 missense variants, 3 splice-site variants, and 2 in-frame deletion variants (Table 2). Most variants (27/28;96.4%) are located in the SAND or adjacent ZnF domain. Only the p. Lys305del variant is in the NLS domain. The *de novo* pathogenic mutations in *DEAF1* caused VSVS to be located in or around the SAND domain. It has been demonstrated that *de novo* pathogenic variants damaged the *DEAF1* transcriptional inhibitory activity of *DEAF1* and reverse *DEAF1*-mediated transcriptional activation at the Eif4g3 promoter (2, 8). A *de novo* heterozygous pathogenic missense variant of the *DEAF1* gene that was located within the SAND domain was detected in our patient. In addition, no candidate genes were detected other than *DEAF1* in this child by WES. It has been reported that a dominant negative effect caused by missense mutations of *DEAF1* genes has been regarded as a possible pathogenic mechanism of VSVS (2, 8). Vulto-van Silfhout and his colleagues put forward that the dominant negative effect of missense mutations in individuals of VSVS could cause severe clinical presentations (8). The in-frame deletion mutations could result in amino acid change (2). The splicing site variations play an extremely important role during the alternative splicing of *DEAF1* mRNA transcriptions (2). Sharma et al. (3) proposed that the splice-site variants disrupt gene function by forming a truncated protein impeding further downstream reactions rather than having a dominant negative effect that leads to relatively less serious clinical manifestations.

**Table 2:** Pathogenic *de novo* variants of *DEAF1* that have been previously reported.

cDNA change	Amino acid change	Protein domain	Mutation type	References
c.670C>T	p. R224W	SAND	Missense	(1) (*n* = 2); (8)(*n* = 1)
c.608 T>C	p. L203P	SAND	Missense	(1) (*n* = 1)
c.825C>T	p. H275Q	SAND-like	Missense	(1) (*n* = 1)
c.634G>A	p. Gly212Ser	SAND	Missense	(2)(*n* = 2); (4)(*n* = 1)
c.637A>C	p. Thr213Pro	SAND	Missense	(2) (*n* = 1)
c.640C>G	p. Leu214Val	SAND	Missense	(2) (*n* = 1)
c.641T>C	p. Leu214Pro	SAND	Missense	(2) (*n* = 1)
c.646A>G	P. Lys216Glu	SAND	Missense	(2) (*n* = 1)
c.648G>T	p. Lys216Asn	SAND	Missense	(2) (*n* = 1)
c.664+1G>T	p.?	SAND	Splice site	(2) (*n* = 1)
c.674G>A	p. Gly225Glu	SAND	Missense	(2) (*n* = 1)
c.683 T>C	p. Ile228Thr	SAND	Missense	(2) (*n* = 1)
c.706A>G	p. Ser236Gly	SAND	Missense	(2) (*n* = 1)
c.757A>G	p. Lys253Glu	SAND	Missense	(2) (*n* = 1)
c.762_764delAAG	p. Arg254del	SAND	Deletion	(2) (*n* = 1)
c.791A>C	p. Gln264Pro	SAND	Missense	(2) (*n* = 1); (4) (*n* = 1); (9) (*n* = 1)
c.815T>C	p. Leu272Ser	SAND	Missense	(2) (*n* = 1)
c.826G>C	p. Ala276Pro	SAND-like	Missense	(2) (*n* = 2)
c.762A>C	p. R254S	SAND	Missense	(2) (*n* = 1)
c.702G>T	p. W234C	SAND	Missense	(2) (*n* = 1)
c.700T>A	p. Trp234Arg	SAND	Missense	(2) (*n* = 1)
c.913_915del	p. Lys305del	NLS	Deletion	(2) (*n* = 1)
c.815_817delinsG	p. Leu272^*^	SAND	Splice site	(3) (*n* = 1)
c.664+2T>G	p.? intronic	SAND	Splice site	(5) (*n* = 1)
c.762A>C	p. Arg254Ser	SAND	Missense	(6) (*n* = 1)
c.737G>C	p. Arg246Thr	SAND	Missense	(7) (*n* = 1)
c.662C>T	p. S221L	SAND	Missense	(15) (*n* = 1)
c.683T>G	p. Ile228Ser	SAND	Missense	(10) (*n* = 1)

The “L272^*^” mutation resulted in a stop codon and premature truncation of the protein at amino acid 272.

What needs to be specifically identified is the recessively inherited dyskinesia, seizures, and intellectual developmental disorder syndrome (DYSEIDD). In terms of clinical findings, with the exception of microcephaly, the clinical features of DYSEIDD and VSVS were basically the same (2). DYSEIDD is caused by the biallelic pathogenic variants in the *DEAF1* gene (MIM 617171) (2). In the *DEAF1* gene mutations, compared to the *de novo* variants, only pathogenic biallelic variants can lead to microcephaly. However, it is regrettable that not all individuals with DYSEIDD have microcephaly (2). Therefore, genetic sequencing, such as WES or whole genome sequencing, is required to distinguish the two diseases.

In summary, our patient and prior reports showed that different AEDs revealed different results in managing seizures in those with VSVS. This issue needs further research. Our patient provides additional information on the phenotype, genotype, and effective therapy in VSVS.

## 5 Conclusion

We described a Chinese VSVS boy with a *de novo* novel pathogenic missense variant of *DEAF1* located within the SAND domain. It enriches the known range of *DEAF1 de novo* pathogenic variants and adds to the few reports of VSVS. In addition, we speculate that recurrent respiratory infections like previously reported sleep problems are not permanent but may improve naturally over time.

## Data availability statement

The datasets presented in this article are not readily available because of ethical and privacy restrictions. Requests to access the datasets should be directed to the corresponding authors.

## Ethics statement

The studies involving human participants were reviewed and approved by the Medical Ethics Committee of Sichuan Provincial Maternity and Child Health Care Hospital (Approval Code:20230331-026). Written informed consent to participate in this study was provided by the participants’ legal guardian/next of kin. Written informed consent was obtained from the individual(s), and minor(s)’ legal guardian/next of kin, for the publication of any potentially identifiable images or data included in this article.

## Author contributions

HZ designed this study, collected and integrated data, and wrote the article. SZ integrated the data. QJ, YP, YH, YC, TH, WD, XL, LZ, and AC participated in the evaluation and treatment of the patient. JW and ZL participated in the study’s design and coordination, revised article. All authors contributed to the article and approved the submitted version.

## References

[1] Chen S Deng X Xiong J He F Yang L Chen B. De novo variants of DEAF1 cause intellectual disability in six Chinese patients. Clin Chim Acta. (2021) 518:17–21. doi: 10.1016/j.cca.2021.02.026, PMID: 33705764

[2] Nabais Sa MJ Jensik PJ McGee SR Parker MJ Lahiri N McNeil EP. De novo and biallelic DEAF1 variants cause a phenotypic spectrum. Genet Med. (2019) 21:2059–69. doi: 10.1038/s41436-019-0473-6, PMID: 30923367

[3] Sharma P Gambhir PS Phadke SR Mandal K. Expanding the phenotype in autosomal dominant mental retardation-24: a novel variation in DEAF1 gene. Clin Dysmorphol. (2019) 28:94–7. doi: 10.1097/MCD.0000000000000252, PMID: 30451703

[4] Chen L Jensik PJ Alaimo JT Walkiewicz M Berger S Roeder E. Functional analysis of novel DEAF1 variants identified through clinical exome sequencing expands DEAF1-associated neurodevelopmental disorder (DAND) phenotype. Hum Mutat. (2017) 38:1774–85. doi: 10.1002/humu.23339, PMID: 28940898 PMC5679464

[5] Li SJ Yu SS Luo HY Li X Rao B Wang Y. Two de novo variations identified by massively parallel sequencing in 13 Chinese families with children diagnosed with autism spectrum disorder. Clin Chim Acta. (2018) 479:144–7. doi: 10.1016/j.cca.2018.01.025, PMID: 29366832

[6] Berger SI Ciccone C Simon KL Malicdan MC Vilboux T Billington C. Exome analysis of Smith-Magenis-like syndrome cohort identifies de novo likely pathogenic variants. Hum Genet. (2017) 136:409–20. doi: 10.1007/s00439-017-1767-x, PMID: 28213671 PMC5848494

[7] Wenger AM Guturu H Bernstein JA Bejerano G. Systematic reanalysis of clinical exome data yields additional diagnoses: implications for providers. Genet Med. (2017) 19:209–14. doi: 10.1038/gim.2016.88, PMID: 27441994

[8] Vulto-van Silfhout AT Rajamanickam S Jensik PJ Vergult S de Rocker N Newhall KJ. Mutations affecting the SAND domain of DEAF1 cause intellectual disability with severe speech impairment and behavioral problems. Am J Hum Genet. (2014) 94:649–61. doi: 10.1016/j.ajhg.2014.03.013, PMID: 24726472 PMC4067565

[9] Rauch A Wieczorek D Graf E Wieland T Endele S Schwarzmayr T. Range of genetic mutations associated with severe non-syndromic sporadic intellectual disability: an exome sequencing study. Lancet. (2012) 380:1674–82. doi: 10.1016/S0140-6736(12)61480-9, PMID: 23020937

[10] Vissers LE de Ligt J Gilissen C Janssen I Steehouwer M de Vries P. A de novo paradigm for mental retardation. Nat Genet. (2010) 42:1109–12. doi: 10.1038/ng.712, PMID: 21076407

[11] Bodunova N Vorontsova M Khatkov I Baranova E Bykova S Degterev D. A unique observation of a patient with Vulto-van Silfhout-de Vries syndrome. Diagnostics. (2022) 12:1887. doi: 10.3390/diagnostics12081887 36010237 PMC9406734

[12] Zhu H Zhao ZH Zhu SY Xiong F He LH Zhang Y. Renal-hepatic-pancreatic dysplasia-1 with a novel NPHP3 genotype: a case report and review of the literature. BMC Pediatr. (2022) 22:603. doi: 10.1186/s12887-022-03659-7, PMID: 36253741 PMC9578240

[13] Richards S Aziz N Bale S Bick D Das S Gastier-Foster J. Standards and guidelines for the interpretation of sequence variants: a joint consensus recommendation of the American College of Medical Genetics and Genomics and the Association for Molecular Pathology. Genet Med. (2015) 17:405–24. doi: 10.1038/gim.2015.30, PMID: 25741868 PMC4544753

[14] Yip L Creusot RJ Pager CT Sarnow P Fathman CG. Reduced DEAF1 function during type 1 diabetes inhibits translation in lymph node stromal cells by suppressing Eif4g3. J Mol Cell Biol. (2013) 5:99–110. doi: 10.1093/jmcb/mjs052, PMID: 22923498 PMC3604916

[15] Barker HE Smyth GK Wettenhall J Ward TA Bath ML Lindeman GJ. Deaf-1 regulates epithelial cell proliferation and side-branching in the mammary gland. BMC Dev Biol. (2008) 8:94. doi: 10.1186/1471-213X-8-94, PMID: 18826651 PMC2570686

[16] Bottomley MJ Collard MW Huggenvik JI Liu Z Gibson TJ Sattler M. The SAND domain structure defines a novel DNA-binding fold in transcriptional regulation[J]. Nat Struct Biol. (2001) 8:626–33. doi: 10.1038/89675, PMID: 11427895

